# Development and validation of the UserInvolve comprehensive toolkit for evaluating co-production in research: A guiding resource for researchers

**DOI:** 10.1186/s40900-025-00759-3

**Published:** 2025-08-06

**Authors:** Anneli Gustafsson, Urban Markström, Hilda Näslund, Petra Svedberg

**Affiliations:** 1 Department of Social Work, Umeå University, Umeå, Sweden; 2 The Swedish Partnership for Mental Health, NSPH, Stockholm, Sweden; 3 School of Health and Welfare, Halmstad University, Halmstad, Sweden

**Keywords:** Mental health, Co-production, Service users, Involvement, Research, Evaluation toolkit, Instrument

## Abstract

**Background:**

Despite the evident trend in health research to emphasise co-production approaches, there is a lack of established, comprehensive and concrete strategies and evaluation methods to effectively guide and assess them. This project aimed to develop, validate, and test a toolkit designed to enhance and evaluate co-productions in mental health research. The toolkit includes practical evaluation tools, such as a structured questionnaire and tailored interview guides, to support the initiation of research projects and assess the involvement, process and impact of co-production efforts.

**Methods:**

This project used a co-production approach with formative research design to develop a comprehensive toolkit for evaluating the process and impact of co-production in mental health research. Conducted between 2022 and 2024, the project involved iterative engagement with diverse stakeholder groups, providing a dynamic testbed for developing, validating, and field-testing the instruments. The paper outlines the four-phase process: (1) toolkit generation, (2) validation, (3) field-testing, and (4) completion, detailing how the co-production approach shaped the toolkit’s design, relevance, usability, and rigor.

**Results:**

The result of this project is a structured, practical, and comprehensive co-production evaluation toolkit designed specifically for mental health research, potentially involving a wide range of partnerships. The toolkit includes a project initiation guide, a process-oriented survey and interview-guide for mid- and post-project evaluations, and an impact-focused post-project group interview guide.

**Conclusions:**

The findings address a critical gap in mental health research by developing a structured, practical, and comprehensive co-production evaluation toolkit. The toolkit offers comprehensive strategies for evaluating involvement and both the processes and impacts of co-production throughout a project’s lifecycle.

**Supplementary Information:**

The online version contains supplementary material available at 10.1186/s40900-025-00759-3.

## Abstract (plain language summary)

There is an evident trend in health research to emphasise co-production approaches, where service users, health care providers and researchers collaborate together, to improve the mental health research. Nevertheless, there is a lack of established methods to guide and evaluate these kinds of co-productions. The aim with the study was to develop, validate and field-test a toolkit that can be used to evaluate co-productions within mental health research, throughout its life cycle.

The first draft of the toolkit was based on a mix of existing evaluation instruments and strategies. These were specified and developed into a toolkit. As a second step, this toolkit was validated by an expert group, including representatives from service user organisations and service user providers/agencies. As a third step, the toolkit was field-tested on various co-produced research projects within a research program, called UserInvolve. As a last step, the collected feedback from the field-tests was analyzed and the toolkit finalized.

This work resulted in a practical evaluation toolkit, including a survey and interview guides. These can be used to support the initiation of a co-produced research project and evaluate the co-production in terms of involvement, process and impacts. The UserInvolve co-production evaluation toolkit can be found at the website https://www.umu.se/forskning/grupper/userinvolve/.

**Supplementary Information:**

The online version contains supplementary material available at 10.1186/s40900-025-00759-3.

## Background

The integration of service user (SU) voices and knowledge within mental health services is fundamental for improving the quality, effectiveness and consistency of the services [1–3]. By actively involving SUs in service delivery, mental health services can become more responsive and inclusive in meeting the diverse needs of individuals seeking support and care [4]. Alongside significant shifts in SU involvement within mental health services, there is an evident trend within the research community towards emphasising strategies for co-production [5, 6]. Co-production in research has been defined as “an umbrella term used to describe the process of generating knowledge through partnerships between researchers and those who will use or benefit from research” [7] and it can occur at the individual, group, or collective level [8]. Co-production in research can improve transparency, facilitate the identification of research questions that are more relevant and meaningful to diverse perspectives and needs, improve the overall quality of the research process [9–11]. A co-production approach in research can also help ensure that mental health services are more effectively tailored to the unique experiences and perspectives of SUs, ultimately leading to improved impact and a more supportive environment for all involved. However, like the challenges encountered in SU involvement, efforts to co-produce research are affected by practical constraints, including disparities in resource allocation, limitations in opportunities for participant engagement, and the attendant risks of co-optation [12].

This evolving landscape highlights the critical need for the development of comprehensive evaluation tools tailored for co-production research initiatives. These tools should not only assess the extent of SU involvement and collaboration, but also measure the effectiveness of these approaches in achieving meaningful impacts for all partners involved. By systematically evaluating co-production processes, mental health research can refine its strategies, address challenges, and ultimately enhance the quality and impact of their collaborative attempts. While previous research has outlined various models of co-production in research, a significant gap remains in defining the concepts core features [8], methods of evaluation [13, 14] and understanding its effectiveness, impact and value [15, 16]. Previous research has specifically pointed out that there is a lack of standardized co-production evaluation approaches with wide consensus in the scientific community [17, 18]. Existing instruments tend to focus narrowly on isolated aspects, such as specific individual experiences or activities, rather than capturing the broader, systemic impacts of co-production [19]. This fragmentation and extensive use of a range of self-developed study-specific measurements hinders the ability to comprehensively assess the processes, outcomes, and long-term value of co-production initiatives in research. To address these gaps, there is a critical need to develop holistic and integrated evaluation frameworks that better reflect the complexity and systemic nature of co-production in research. Such frameworks need to move beyond event-based models—such as those relying on individual workshops or interviews where different actors participate only at specific points in time—and instead emphasize continuous engagement and the cumulative impact of collaborative efforts. This underlines the need for concrete validated evaluation instruments, which can be used for research projects that consistently work with co-production methods and multiple partnerships, from planning to post-project phase.

### Aim and purpose

The aim of this paper is to describe the development, validation and field-testing of a set of evaluation instruments – a comprehensive toolkit designed to enhance and evaluate co-production in research within mental health settings. This article also introduces this toolkit, featuring practical evaluation tools, such as a structured questionnaire and tailored interview guides. These tools are designed to support the initiation of research projects and the evaluation of involvement, process and impact of co-production efforts. By providing a clear and transparent description of this formative process of developing, validating and field-testing the evaluation toolkit, this paper is intended to serve as a guiding resource for researchers, SUs and practitioners, offering valuable insights and methodologies to support the evaluation of co-production initiatives. 

### The setting

The UserInvolve research program served as the setting for developing and field-testing the co-production evaluation toolkit. Within UserInvolve, co-production is understood as a collaborative research approach in which knowledge is generated through equitable partnerships between researchers and those intended to use or be affected by the research—primarily service users, but also practitioners and other relevant stakeholders. This approach involves the active engagement of these actors throughout the research process, aiming to integrate multiple forms of expertise in order to produce knowledge that is contextually grounded and practically relevant. Rather than positioning researchers as the sole knowledge producers, co-production emphasizes shared ownership of both the research process and its outcomes. The program specifically focuses on strengthening SU involvement across individual, organizational, and systemic levels in Sweden’s mental health sector [20]. A distinctive feature of the Swedish context is the strong collaboration between the public sector and the SU movement, which plays a leading role in democratizing welfare services and advancing user involvement methods [21]. The Swedish Partnership for Mental Health (NSPH), a national umbrella organization for SU associations, is a key partner in the UserInvolve program, with two NSPH-employed coordinators facilitating activities. UserInvolve comprises several empirical sub-projects, each testing specific co-production methods such as peer support, user-focused monitoring, shared decision-making, and systemic-level SU involvement. These sub-projects, each with their own co-production teams, serve as real-world test-environments for developing and refining the evaluation toolkit [20].

## Method

**Fig. 1:**
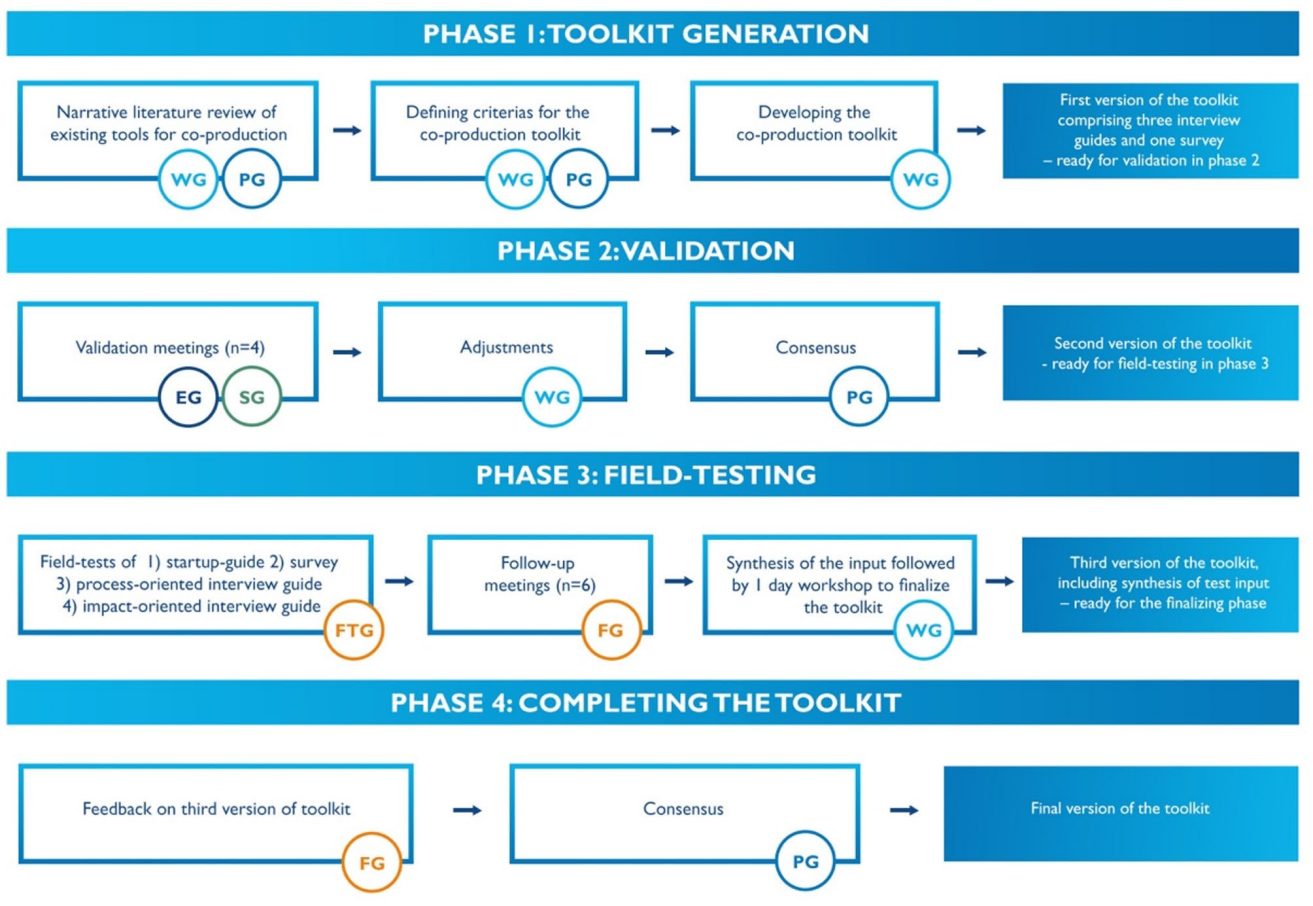
The co-production process in four phases for developing the UserInvolve evaluation toolkit. WG – Working group, including selected researchers (*n* = 3), coordinator from SU organization who is also a researcher (*n* = 1) and coordinator from SU organization (*n* = 1). PG - The UserInvolve program group, including all researchers (*n* = 12), coordinator from SU organization who is also a researcher (*n* = 1) and coordinator from SU organization (*n* = 1). SG - The UserInvolve strategy group, including SU organization representatives (*n* = 3), community based social support services representatives (*n* = 1), health care provider representative (*n* = 1), national health care agencies representative (*n* = 6). EG – Expert group, including service user representative, service provider representative, service agency representative (*n* = 5). FTG – Field-tested groups (*n* = 6). A more detailed explanation of these groups and included participants can be found in the field-testing section of the methodology description. FG – Field-testing groups (*n* = 5), including the UserInvolve SU coordinators, moderator for the interviews and project leading researchers from the various empirical UserInvolve sub-projects

In this project, a co-production approach was employed aiming to develop a comprehensive toolkit for evaluating involvement, process and impact of co-production in research within mental health settings. This co-production approach was designed as a formative study, inspired by McMeekin et al.’s model which emphasizes iterative stakeholder engagement in the development of methodological frameworks [22]. The model outlines key phases in framework development: identifying evidence to inform methodological framework, developing the methodological framework and to evaluating and refining it [22]. Guided by the structure, the study adopted a dynamic, participatory and formative research process between 2022 and 2024, involving iterative, active engagement with diverse stakeholder groups (see Fig. 1). These iterative interactions provided a dynamic test environment for the development, validation and field-test of the toolkit. This paper systematically describes a four-phase framework underpinning this work (Fig. 1): (1) toolkit generation, (2) validation (3) field-testing (4) completing the toolkit. Each phase is described in depth, depicting both the methods employed and the results achieved, illustrating how the co-production approach shaped the toolkit’s design and its relevance and rigor. The reporting is structured according to GRIPP2 (Guidance for Reporting Involvement of Patients and the Public, Version 2, Short Form), an internationally recognized, evidence-based and consensus-informed framework for reporting patient and public involvement in research (Supplementary Material 1) [23].

The toolkit is considered to be the main outcome of this co-produced research. In the process of developing the toolkit, various representatives from the SU movement and the health care sector have been involved, as illustrated (Fig. 1). However, a smaller group of authors have been working with the reporting of the research process and its results. This group involved one representative from the collective SU movement (AG). She is employed by NSPH as one of the SU coordinators in UserInvolve. Two years into the research process she was also employed part time as a postdoctoral fellow at the Department of Social Work at Umeå University.

### Phase 1 – Toolkit generation

The first phase aimed to develop an initial version of the toolkit. As part of this development phase, an approach inspired by narrative literature review methodology was used [24, 25] to identify existing instruments for evaluating co-production within mental health research. This approach aligned well with the formative research design, which focuses on contextual understanding over comprehensive evidence synthesis. Searches in both scientific literature (e.g., PubMed, PsycINFO, Scopus) and grey literature sources were conducted. Concurrently, the research team held several workshops to discuss the instruments identified from the literature. These workshops, alongside extensive discussions within both the working group (WG) and the program group (PG), helped clarify the purpose, content, and objectives of the toolkit. These discussions facilitated the creation of a set of criteria that the toolkit needed to fulfill, laying the foundation for its development. Throughout this process, a preliminary toolkit was developed, comprising two interview guides to be used at mid-point and post-project, as well as a survey for these same stages. Additionally, a project initiation guide was created, which was a modified version of the mid-point interview guide, with questions rephrased in the future tense and some minor adjustments. The toolkit draft was iteratively discussed several times between the WG and the PG.

### Phase 2 – Validation

The second phase focused on validating the preliminary toolkit in terms of both face and content validity [26]. Face validity refers to the clarity and readability of the items/questions and content validity to the adequacy of the tools’ ability to consider what it is presumed to evaluate i.e. if it provides the necessary questions for investigating the content within co-production [26]. The validation aims to ensure that the questions are not only clear and relevant but also gather the necessary data to fulfil the research objectives.

To validate the questions and the content within the interview guides and the survey, an expert group (EG) assembled through purposive sampling [27], comprising SU representatives from different SU-run associations and health care provider/agency representatives. All with extensive expertise and experience in participating in co-produced research. Additionally, the UserInvolve strategy group (SG), which included representatives from SU-run associations, public health agencies, and regional authorities, was involved.

The data collection with the EG took place digitally on three occasions. The UserInvolve SU coordinators from NSPH moderated these sessions and took field-notes. Each meeting lasted two hours and revolved around questions connected to face and content validity in the survey, as well as the process-oriented and the impact-oriented interview guides. The project initiation guide was excluded from this process, due to its similarity to the process-oriented interview guide. During the meetings the group reviewed the instruments within the toolkit. Based on the result of this validation with the EG and the input from the SG, recorded through field-notes as data, the iterative process continued within the WG and the PG, aiming to refine, and finalize the toolkit for the testing phase.

### Phase 3 – Field-testing

Evaluating or piloting the methodological framework is an important step in the development process [22]. The rationale for incorporating field-testing during this formative stage was to examine how the toolkit functioned across diverse project contexts and identify areas for refinement based on real user experiences, thereby laying the groundwork for future formal validation. Hence, in the third phase we field-tested the toolkit through the lense of the criteria that had been established during the toolkit generation phase. The survey and preliminary interview guides were tested in six empirical co-production groups within different projects in the UserInvolve research program, involving different settings of researchers, SU- and health- or care providers/agency representatives (Table 1). These groups were selected to test the different instruments within the toolkit based on which stage they were at in their research processes. The project initiation guide was only tested on one group, due to the fact that all other groups had already started their research process by the time we had a field-testable version of the guide. The survey and the process-oriented interview guide were field-tested five times in projects that were at mid-point, and the survey was tested at one time in a project that had reached the post-project phase. When the toolkit field-testing phase was finished, the research program only had two projects that had ended. Consequently, the impact-oriented interview guide was only tested twice.

**Table 1:** Overview of Co-production groups* and instruments used in field-testing

Instrument	Field-testing groups (FTG)	Participants (*n*)	Participant Description
Project initiation guide	Peer support (subproject 1)	8	Researcher (*n* = 2), SU-representative (*n* = 6)
Survey, mid-point	The UserInvolve strategy group (SG)	17	Researcher (*n* = 5), SU-representative (*n* = 5), health or care provider/agency representative (*n* = 7)
	User-focused monitoring	11	Researcher (*n* = 2), SU-representative (*n* = 8), health or care provider/agency representative (*n* = 1)
	Peer support (subproject 1)	7	Researcher (*n* = 1), SU-representative (*n* = 6)
	Peer support (subproject 2)	10	Researcher (*n* = 2), SU-representative (*n* = 6), health or care provider/agency representative (*n* = 2)
Survey, post-project	User-focused monitoring	8	Researcher (*n* = 3), SU-representative (*n* = 4), health or care provider/agency representative (*n* = 1)
Process-oriented interview guide	The UserInvolve strategy group (SG)	12	Researcher (*n* = 3), SU-representative (*n* = 4), health or care provider/agency representatives (*n* = 5)
	User-focused monitoring	8	Researcher (*n* = 3), SU-representative (*n* = 4), health or care provider/agency representative (*n* = 1)
	User involvement at systemic level	11	Researcher (*n* = 3), SU-representative (*n* = 7), health or care provider/agency representative (*n* = 1)
	Peer support (subproject 1)	5	Researcher (*n* = 2), SU-representative (*n* = 3)
	Peer support (subproject 2)	7	Researchers (*n* = 2), SU-representative (*n* = 4), health or care provider/agency representative (*n* = 1)
Impact-oriented interview guide	Shared decision making in coordinated individual plans	5	Researcher (*n* = 2), health or care provider/agency representative (*n* = 3)
	User-focused monitoring	8	Researcher (*n* = 3), SU-representative (*n* = 5)

*These groups are further described in Markström et al. 2023 [20]

The data collected for the field-tests was survey response, group interviews and follow-up meetings. The survey data consisted of the two final statements added to the survey, which addressed whether the questions were relevant for evaluating co-production and whether the survey was easy to complete. In addition, participants were given the opportunity to provide open-ended comments if there was anything further, they wished to convey to the researchers. The interviews were conducted and recorded digitally and thereafter transcribed. For both the process-oriented and the impact-oriented field-test interviews, two moderators (researchers within the UserInvolve program who were not part of the sub-project currently under evaluation) conducted the interviews. Everyone within the project group, including researchers and all partners, were participants during the interview. After these activities, five follow-up digital meetings were conducted with field-testing groups (FG). These meetings where recorded and transcribed. The primary objectives of these meetings were to gather feedback on the sequence of questions, potential ambiguities, missing or unnecessary themes, follow-up questions, and the overall frame of the toolkit [28].

After synthesizing the input from the field-testing phase, the WG conducted an internal workshop to analyze the data. This analysis followed a directed qualitative content analysis approach [29] using the criteria established in Phase 1. Through this process, any needed adjustments within the toolkit were identified to meet its intended objectives.

### Phase 4 – Completing the toolkit

The fourth phase entailed, as suggested by McMeekin et al., revising the methodological framework to incorporate modifications identified during the field-test phase [22]. The aim of this phase was to present a clear, logical, and complete toolkit to use in real-world settings [30]. The third version of the toolkit was sent to the FG for written suggestions and adjustments. The WG then finalized a fourth version of the toolkit for the PG to reach consensus on.

## Results

### Phase 1 - Toolkit generation

During the first phase, WG and PG searched in both scientific and grey literature for instruments, models and strategies that could meet the evaluation needs and purposes of the research program. A framework was needed to facilitate various evaluation strategies at different stages of the research process. This framework also needed to be user-friendly and transparent, enabling effective tracking of co-production research projects and their impacts from start to finish. A useful literature review by Greenhalgh et al. of existing framworks for patient and public involvement in research was found [31].

Existing instruments and models were discussed in WG and PG through the lens of the co-production design within the UserInvolve research program, in combination with previous experiences from co-produced research within the research group. Several existing instruments that were identified primarily emphasized individual experiences, prioritizing the SU perspective, while paying less attention to the broader dynamics and significance of co-production processes within research projects [13, 15, 17]. In addition, the different phases of co-production, and their respective roles and impacts, are frequently overlooked in existing evaluations [32]. For the purpose of UserInvolve, it was essential to adopting a more holistic and collective approach, one that acknowledges co-production as a collective, multi-actor process unfolding across different phases, and integrates the perspectives and contributions of service users, practitioners, and researchers.

**Fig. 2:**
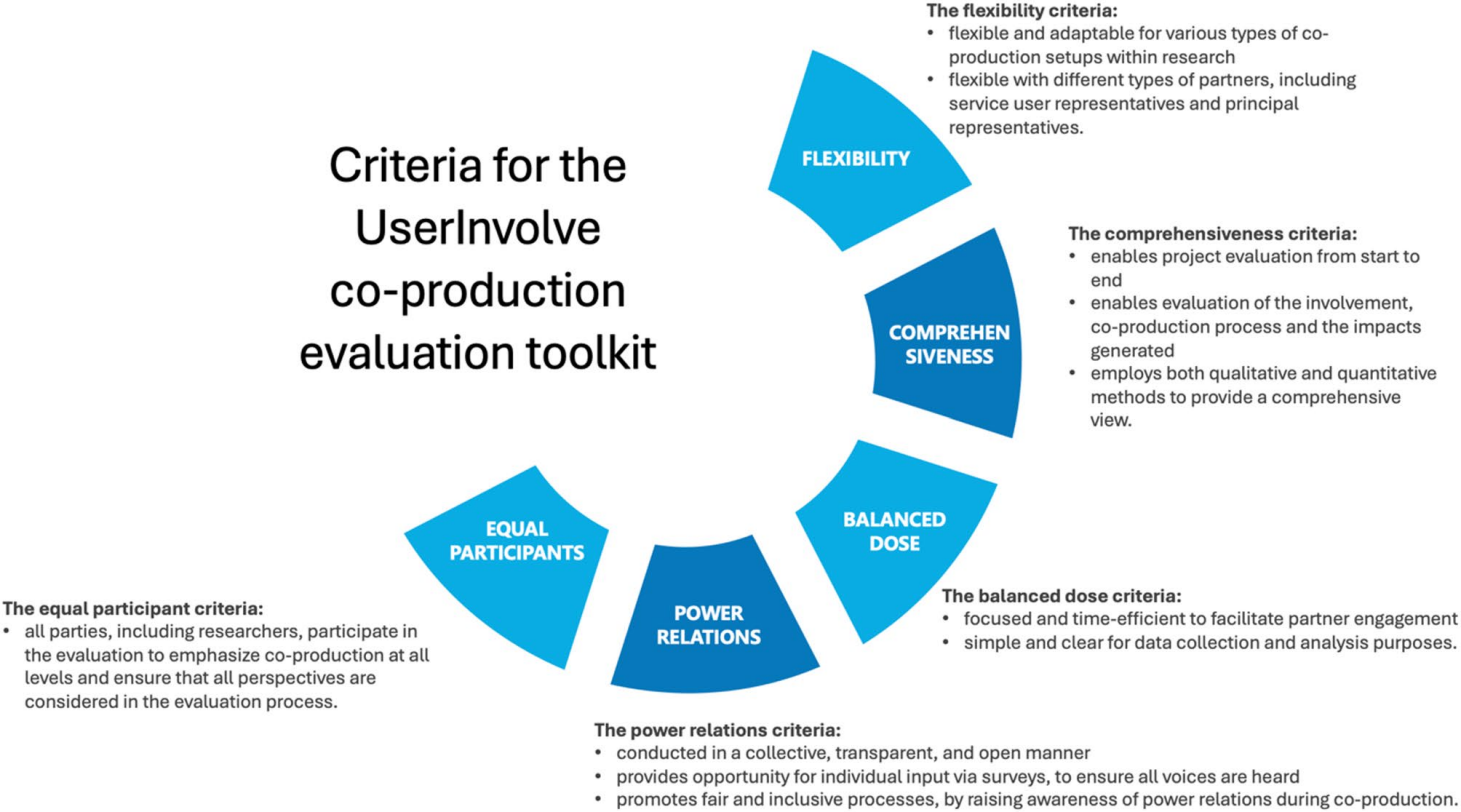
Criteria for the UserInvolve co-production toolkit

Building on this insights, the iterative process of reviewing existing instrument and strategies enabled the WG and PG to further define a set of criteria that the methodological framework had to fulfil (Fig. 2). Defining clear criteria for co-production was a critical step in the development of the framework. This was essential to ensure that the process moved beyond tokenistic inclusion, where stakeholders are present but lack meaningful influence, and instead embodied a genuine co-production approach. Thus, these criteria indicated the necessity for the toolkit to be comprehensive, appropriately balanced and flexible, effectively supporting and evaluating co-production within research projects, while ensuring fair and inclusive processes. The criteria also guided the selection and integration of individual pre-existing instruments, which were subsequently refined into a cohesive toolkit for ongoing use.

In parallell with this work, the PG also identified three critical dimensions that were essential to include in the evaluation strategies of co-production in research: involvement-oriented evaluation, process-oriented evaluation, and impact-oriented evaluation. The rationale for adding the dimensions was that each of them address different but equally important aspects of the co-production process and collectively provide a comprehensive framework for evaluation. One could argue that involvement must be integrated in the process- and impact-oriented evaluation dimensions. However, had involvement not been emphasized as a distinct dimension, it might paradoxically have been at risk of being subordinated to the process- and impact-oriented dimensions.

In response to the identified need to emphasize evaluation from an involvement-oriented perspective, both the WG and the PG drew inspiration from the Involvement matrix developed by Smits et al. [33], which is an already existing conversation instrument visualised in the form of a matrix. The advantage with this matrix is its ability to articulate five possible roles in a co-productive research project i.e., Listener, Co-thinker, Advisor, Partner, and Decision-maker, during three research phases i.e., Preparation, Execution, and Implementation. This enables discussions between researchers and co-producing partners concerning what roles of involvement they wish to take, or have taken, in different phases of the research project. The WG added research-specific sub-categories to the matrix phases and introduced a new phase, ‘Communication of Results,’ to emphasize the importance of effectively sharing research findings. This addition aims to ensure all partners can utilize the results, thereby enhancing the overall impact and reach of the research.

For the development of a process-oriented evaluation tool the WG and the PG was inspired by an already existing tool, namely The Patient Engagement Quality Guidance Tool [34], due to its ability to point out meaningful elements within a co-production research process, such as shared purpose, respect and accessibility, representativeness of stakeholders, roles and responsibilities, capacity and capability for engagement, communication and documentation, continuity and sustainability. Drawing from these elements, the WG developed questions for the survey and interview guides for project initiation and mid-point evaluations. The survey and interview guides included the same set of questions to ensure consistency. The survey and the mid-point group interview guide were designed to be used in combination in the process-oriented evaluation within the toolkit. The rationale for using both methods was to ensure capturing individual and anonymous experiences via survey, and the collectively formulated experiences through the interview guide, providing deeper insights and enriching the data from the survey. The interview guide for the project initiation was not intended as a baseline evaluation. Instead, it was designed to facilitate a guided conversation where researchers and their co-production partners could meet and discuss key questions about how they should collaborate in a co-production manner. This approach allowed for establishing a mutual understanding of roles, responsibilities, and expectations.

Furthermore, an impact-oriented evaluation tool was needed to be used in a post-project phase. Here the WG and the PG found inspiration from the Social model of impact [32], an already existing model which aim is to deepen the understanding of impacts that the research has generated, as well as the central mechanisms that have enabled these impacts. The model includes five different impact levels: individual, group, organisational, societal, and paradigmatic. The WG created a group interview guide that included questions related to each level relevant for the mental health context.

The main outcomes of Phase 1 included a preliminary toolkit comprising a project initiation guide, a process-oriented survey for mid-point and post-project stages, a process-oriented interview guide for mid-point evaluation, and an impact-oriented interview guide for post-project evaluation. This toolkit is applied in Phases 2 and 3.

### Phase 2- Validation

The validation of the evaluation instruments included in the co-production research toolkit— a process-oriented survey, a process-oriented group interview guide and an impact-oriented group interview guide —yielded overall satisfactory results concerning both face and content validity. The toolkit was found to be relevant and appropriate for evaluating co-production in research. Participants recognized the relevance of the items and confirmed the content validity of the tools, specifically, their adequacy in capturing key aspects of co-production. This suggests that the toolkit includes the necessary and meaningful questions to support the investigation of co-production processes within research settings. However, as illustrated in the validation process fieldnotes below, the WG also received constructive feedback that included redefining certain themes and questions to better capture partners experiences, adding specific elements to address overlooked aspects of the co-production process, and improving the clarity and comprehensiveness of the survey and interview guides. Field-notes from an EG meeting exemplifies how and why themes were rephrased: *“The meaning of responsibility in the theme Roles and responsibilities was considered unclear in this context and was suggested to be changed to ‘opportunity to influence*,*’ since two out of three questions in the original survey dealt with the extent to which one had been able to influence”.*

Additionally, there was a strong emphasis on the importance of understanding participant motivations, as stated in field-notes from EG meeting: *“This was considered important because responses to such a question were seen as potentially providing valuable insights into why participants chose to engage in the co-production process.”* It also became apparent that the number of questions in the interview guide needed to be streamlined and prioritized, and all survey questions converted into statements to align with the Likert scale format. WG synthesized all the feedback and refined the toolkit further to ensure it effectively meets the needs of all partners involved (Supplementary Material 2).

In addition to all the detailed input to the different instruments within the toolkit, significant suggestions regarding interview context for both the process- and the impact-oriented interview guides were brought forward, as observed in the fieldnote: “*The user experts in particular raised power dynamics related to the ‘setup’ of the focus group interview. There needs to be awareness that it can be difficult for some participants to speak openly when the researchers leading the project are present alongside all other participants in the conversation. However*,* arguments were also raised emphasizing the importance of everyone being present*,* since this is a matter of co-production.”*

EG engaged in intensive discussions concerning the structure of group interviews and they ultimately recommended having two moderators present during group interviews, with at least one possessing lived experience of mental health challenges, to address potential concerns related to self-stigma. It was further suggested that both moderators should be external to the project. Nonetheless, concerns were voiced by SG regarding the feasibility of using moderators unfamiliar with the project context. “*The role of the moderator was highlighted as important during group interviews. While having a neutral person can be beneficial*,* there is a risk that much of what has been done may be overlooked if the moderator is entirely external.”* As a solution, it was decided that PG (including many researchers working in different sub-projects within UserInvolve), some of whom openly shared their own experiences, would serve as a moderator pool.

### Phase 3 – Field-testing

The results identified areas where the toolkit aligned with or diverged from the established criteria, offering insights that informed the adjustments to enhance its overall effectiveness. Detailed descriptions of this phase are found in Supplementary Material 3.

### The equal participants’ criteria

During field-testing, the equal participants’ criteria encountered several challenges, particularly regarding the role of the researcher. This was evident in the use of all tools.

In the survey, the field-testing highlighted the absence of a question identifying the participant’s role as SU representative, service provider/agency representative, researcher, or other. It became clear that participants needed the option to select multiple roles when applicable.

In the project initiation guide, the researchers expressed discomfort with the way the questions were framed. To better facilitate project initiation conversations between equals, adjustments were made to present the guide as a set of themes that researchers could use to initiate more open-ended discussions. As one researcher expressed it: “*emphasize that this is not an interview*,* but rather a joint conversation between the researchers and the partners. To establish a foundation for collaborating in the best possible way. And to build that trust”.* Sub-questions were restructured into suggested sub-themes instead of direct interview questions. Additionally, instructions were modified to emphasize that the primary objective of the project initiation meeting is to foster a collective sense of “we” for the upcoming co-production process.

The field-testing phase revealed that researchers often struggled to avoid taking a leading role in conversations. Their expertise and familiarity with the subject often led them to provide additional commentary or guide discussions toward critical thinking towards the research group. As one researcher puts it: “*I also think it helps build trust — that they get to hear how we’re genuinely attentive to the risk of tokenism and that we maintain this open dialogue.”* Moreover, researchers who initiated the project and assumed leadership roles found it challenging to respond to the Involvement matrix. Questions about role definition were more relevant for the project partners than to the researchers themselves, making it difficult for the researchers to answer effectively. However, the WG and the PG found that it remains important for the researchers to engage in discussions about their partners roles during the research process.

### The power relations criteria

Concern was raised whether the participants could feel comfortable expressing critical views when project leading researchers were present during the group interviews. It was acknowledged that ongoing negotiations between researchers, partners, and their organizations might lead participants to respond strategically, potentially filtering or censoring their feedback. One researcher expressed it: *“During this interview*,* you step into the role of a strategic person. None of us really speak completely from the heart*,* so to speak—we all censor ourselves”.* While this is a valid concern, field-tests did not persuade us to alter our approach. Excluding researchers would diverge from the co-production process, and conducting only individual interviews with all participants could risk overloading the data collection and upsetting the balance. To address these challenges, an opportunity for the partners to provide anonymous and critical feedback was enabled in the survey. Additionally, researcher mentioned that they encouraged open criticism by being self-critical themselves when feedback during group interviews was predominantly positive. *“I don’t think I’ve contributed a single comment that was purely positive and supportive. Instead*,* I’ve seen it as my role*,* to some extent*,* to do everything I can to identify aspects that are more critical or problematic”* said one of them.

Ensuring that the research project’s purpose, accessibility, and the implications of co-production for each partner were collectively discussed appeared essential. It was evident during the project initiation that creating a safe and confident environment within the co-production group was a primary focus. It became apparent that in some projects, and regarding all phases (project initiation, mid-point and post-project), the purpose had been predetermined without the involvement of all partners in the initial stage. While this does not necessarily indicate a deficiency in co-production, participants stressed the necessity of continuously revisiting and discussing the research project’s purpose with all partners throughout its progression. This is illustrated by a researcher: *“Regarding the purpose*,* if you’re in a situation like in most research projects*,* where you had to define a purpose already in the funding application*,* then you’re kind of stuck with that purpose. In the best case*,* it’s been co-produced from the beginning… Still*,* you can at least say: this is the purpose as it’s written. That’s one way to approach it. Do you have any thoughts on that? Does it work for you? Does it feel meaningful?”*

Another significant insight was the effectiveness of the Involvement matrix in stimulating discussions about different levels of co-productions through the different research phases. This tool was especially valuable for project groups involved from the start. The field-testing also emphasized the benefits of the matrix’s inherent non-hierarchical approach to different levels of participation. A participant might prefer to be a listener at one stage of the research process, and a partner at another. As one SU representative reflected: *“I think the matrix was a bit of an eye-opener in that regard*,* it got me thinking. That maybe it’s completely logical that I should try to take on or have different roles in different parts*,* where we distribute responsibility. So it’s not about having just one role*,* but rather it depends on what we’re currently trying to achieve and where we are in the process… That’s why I think this whole conversation has been very rewarding*,* because it has shed light on both the project and the phenomenon of co-production from many different and relevant perspectives.”* Additionally, the field-testing highlighted the matrix’s potential as a baseline measure; participants could fill it out at the start of the project and revisit it at mid-project and post-project stages to track and discuss changes in involvement during subsequent group interviews. It was also suggested that the Involvement matrix could be helpful in the project initiation phase for the co-production partners to agree on the level of time commitment with their organization.

### The balanced dose criteria

Field-testing confirmed that mid-point and post-project measures, along with the initial project initiation conversations, were adequate for capturing a comprehensive understanding of the co-production process and its impacts, effectively meeting the balanced dose criteria.

However, the impact-oriented interview guide presented challenges in meeting the balanced dose criteria, as participants often addressed multiple questions in one response, resulting in unstructured and repetitive interviews. Field-tests, in turn, revealed that the questions structured around individual, organizational, and societal levels functioned more as analytical tools than operational ones and were too abstract to be used as direct questions. One of the moderators expressed it like: *“The interview guide was not organized in a sufficient way*,* with the levels of impact, when participants often addressed multiple levels of value in a single answer. It didn’t work very well”*. To simplify usage, the interview guide was revised, inspired by Edwards & Meagher [35], to focus on three main types of impacts from co-produced research: (1) relational, (2) instrumental, and (3) conceptual and cultural. To meet the balanced dose criteria, we streamlined the approach by concentrating probing questions on potential impacts at all levels. The revised interview guide was field-tested in a new setting with positive result, as a moderator stated: “*From an overall perspective*,* I think it created opportunities for conversation and that they wanted to share and talk. [...] And I think the questions worked very well. I also imagine that the terms we used—conceptual*,* cultural*,* and instrumental—are quite difficult. But you introduced it very well*,* (name of the other moderator). You started by talking about the content without using those terms*,* and I think that was really good*,* because otherwise it might have been confusing.”*

### The comprehensiveness criteria

Overall, field-testing confirmed that the toolkit comprehensively evaluates involvement, the research process, and the impacts generated by the research. The combination of qualitative and quantitative measures within the toolkit sufficiently met the comprehensiveness criteria. However, the field-test highlighted the challenges in finding an optimal time for the project initiation conversation, where all partners could participate equally in decision-making regarding the co-production process. One researcher explained it: *“But it’s also one of those questions about time… These are really important issues*,* but you are sitting with… practical questions*,* that you want to bring in. But then you are also thinking about their time*,* like ‘now we’re going to talk about the structure again’”.* Project initiation meetings require some prior planning and decisions, leading to certain partners being more involved than others.

Moreover, based on the FG meetings the WG and the PG found that conducting the process-oriented interview when approaching mid-point was beneficial, as it provided an opportunity to address any issues and reinforce what was working well. The combination of the survey and the in-depth process-oriented group interview proved advantageous. As a moderator states: “*That aspect of co-production*,* where the process of completing a survey*,* engaging in discussion*,* being interviewed*,* and reflecting*,* can itself prove to be an important part of a co-productive process. But there weren’t many of us who initially wished we had made all this concrete and clear from the start. It was more like—it’s really good that we’re taking this pause now. It’s good that we’ve taken the time to stop and reflect at this point.”*

The survey allowed partners to express potential criticism anonymously, while the group interview enabled moderators to focus on questions where survey responses varied significantly, fostering group discussion. Conducting a follow-up survey at the project’s end could further complement the mid-point measurement.

The revised version of the impact-oriented interview guide was tested only at the project’s conclusion. It may be more valuable, however, to conduct this type of group interview after a longer time post- completion, allowing subjective as well as objective impacts to become more apparent. In practice, researchers often move on to other projects and tasks after the final reporting, leaving limited time and capacity for further follow-up.

### The flexibility criteria

Regarding the flexibility criteria, we noticed that the instruments were most suitable for evaluating projects involving a co-production group that remained consistent from start to post-project, maintaining the same partners throughout. We also noted that even within long-term groups, partner representation sometimes shifted due to changes or time constraints within the partners’ organizations, which could make it difficult for some participants to answer certain questions comprehensively. As one SU-representative writes in the survey: *“My role in the project has shifted over time*,* which means I am not able to answer all of the questions”.* At the same time this same person replies “Strongly agree” both when asked if the survey was easy to complete and if the questions were relevant for evaluating co-production.

The variability in how long partners had been involved in the project led some participants to express uncertainty about how their answers might be interpreted, particularly when indicating limited influence over project purpose, as exemplified by a survey response from another SU representative: *“Regarding the question of influencing the purpose of the project*,* my answer is no. This is not because you have not been receptive*,* but because (name of the organization that the person represents) joined the project at a later stage than NSPH*,* which was the organization involved in developing the project’s purpose and goals.*” Acknowledging these challenges is crucial when analyzing data produced using the toolkit. Furthermore, when distributing the survey, it is important to inform participants about the opportunity for them to elaborate on their answers during the process-oriented group interview.

The group interviews were estimated to take about two hours, which the WG and the PG recognized might not be suitable for all service user- or service provider/agency representatives. Planning breaks with participants at the beginning of the interview was necessary. Despite the potential length of these meetings, field-tests indicated that many participants appreciated the interview themes and valued the conversations. A similar response was observed for the survey.

### Phase 4 – Completing the toolkit

**Fig. 3:**
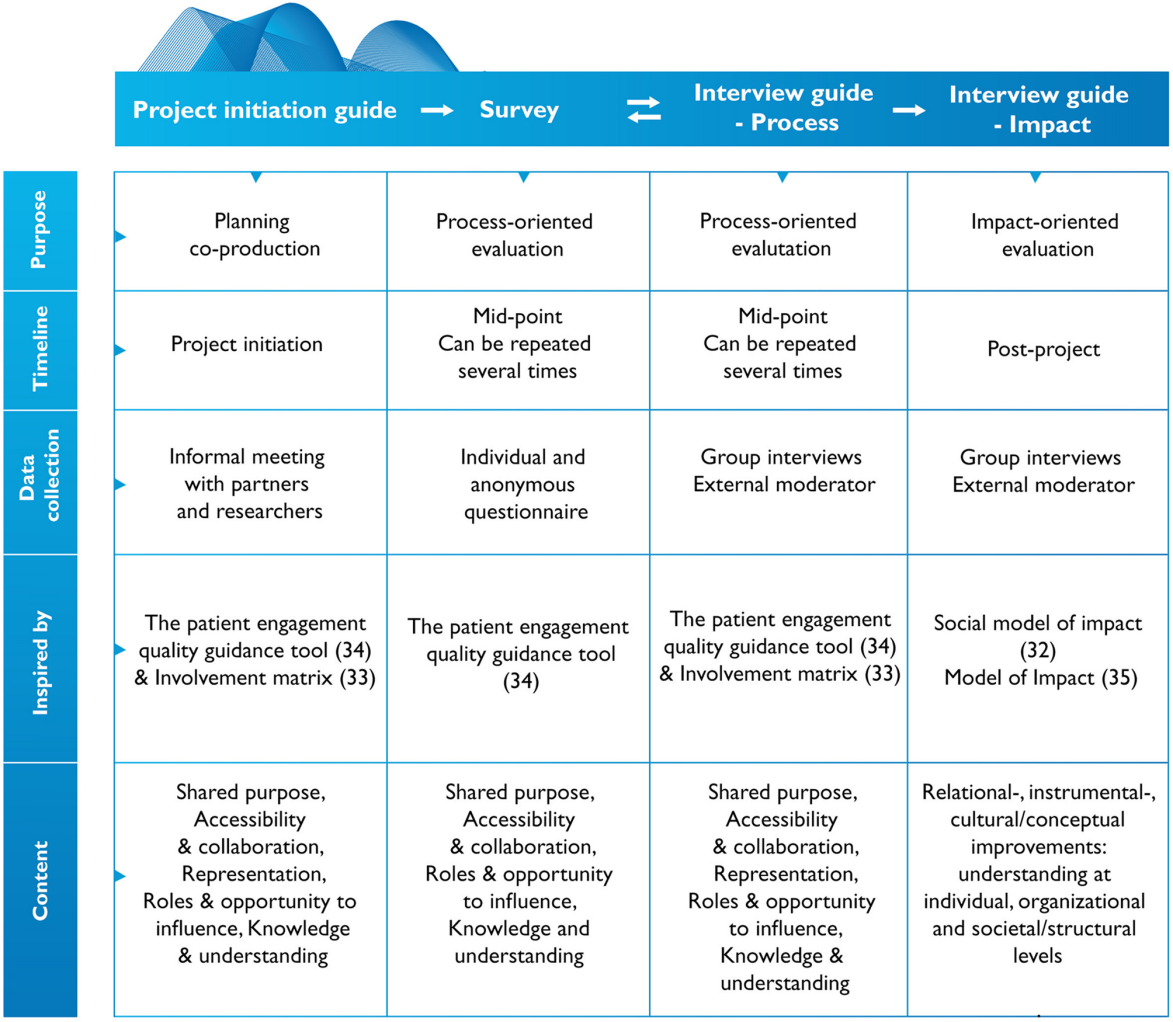
The completed UserInvolve evaluation toolkit

During the fourth and last phase, the WG completed the toolkit with minor adjustments on each instrument based on the input from the field-testing (Supplementary Material 3) and finally reached consensus within the PG on the wholeness of the toolkit. The toolkit was then translated from Swedish into English. First, Copilot and AI-powered assistant was used for translating each instrument within the toolkit. As a second step, these translated instruments were reviewed in relation to the original instrument, by a native English speaker who is also fluent in Swedish. As a final step, the WG discussed the reviewers’ comments and adjustments to ensure that the translated toolkit conformed to the original toolkit. The overall result from the toolkit generation-, validation-, and field-testing phase is the UserInvolve co-production evaluation toolkit, as illustrated above (Fig. 3) and Supplementary Material 4, 5, 6 and 7 for each instrument within the toolkit.

## Discussion

This study addressed the absence of thorough strategies for evaluating involvement, processes and impacts of diverse co-production designs in mental health research, resulting in a structured and comprehensive co-production evaluation toolkit, ready to be used in mental health research potentially involving a wide range of partnerships. The main contribution is the integration and development of existing methods and approaches into a streamlined, validated and field-tested toolkit, aligned with the co-produced criteria developed in phase 1 to address multiple evaluation needs throughout the lifecycle of a co-produced research project. This work contributes to the need for instruments [32] to support and evaluate distribution of roles, levels of involvement and impact throughout the research process, from design to dissemination.

The current study was conducted within the UserInvolve research program, consisting of multiple co-produced research projects and a co-production-based organization monitoring and managing the program [20]. This context provided the development process with a unique test environment with various partners—including SU representatives, health or care provider/agency representatives and researchers—who have influenced the design and the development of the toolkit significantly. The diverse range of perspectives contributed to the creation of a validated and more comprehensive, contextually relevant and responsive toolkit for evaluating co-production throughout the research lifecycle. This toolkit addresses the need for a more explicit evaluation of co-production to fully understand how involvement, process and impacts of engagement are achieved. Addressing this gap is critical to ensure that engagement efforts lead to meaningful and measurable outcomes and impact [36, 37].

In this study, face and content validity, complemented by field-testing, were found to be appropriate methods for developing the toolkit during its formative stage. Both the benefits and challenges of this design are acknowledged. A key learning was the importance of creating structure within the unpredictable and, thus, very complex nature of this research design. It was difficult to foresee all the required steps and how to engage the different co-production groups, and in fact, the formative research design developed along the way. The considerable amount of input regarding each instrument during the validation and field-testing phases had to be registered and managed carefully while ensuring consistency across the toolkit. This was a very demanding and time-consuming process.

On the other hand, the partners feedback and insights during the validation and field-testing phases helped design the tools to become more user-friendly and tailored in capturing the nuances of co-production in research. For instance, they pointed out the importance of adapting questions to be relevant across different research project stages, which led to more refined and targeted questions and sub-questions. Nevertheless, within the framework of formative research, face and content validation combined with field-testing offered a meaningful and pragmatic way to demonstrate the toolkit’s relevance, acceptability, and early utility before potential future steps toward more rigorous summative and psychometric evaluation.

The partners’ involvement shed light on the challenges of roles, balancing representation and maintaining consistency in co-production groups. These issues are explicitly discussed in the literature, which highlights the power imbalances being deeply embedded in research and mental health care systems, often leading to tokenistic engagement [38–40]. Consequently, full representation and equity in co-production remain challenging to achieve. This toolkit development project showed that it was important to constantly discuss expectations related to roles, involvement, and responsibilities to ensure meaningful participation and avoid tokenism.

Another key learning, from the field-testing phase, was the importance of continuously highlighting themes (in the survey and interview guides) —such as shared purpose, roles, accessibility, and cooperation—in joint discussions with all research partners. Co-production extends beyond gathering input on issues and data; it is fundamentally about building relationships and fostering mutual trust [41]. However, our experience from this project is that co-production processes often become packed with agenda items, leaving little time for relationship-building, which is a critical component of successful collaboration. To address this, a dynamic, systematic, and collaborative approach to defining roles in the co-production process can foster meaningful engagement and enhance outcomes [39]. Such an approach is particularly valuable as co-production processes naturally evolve over time, and participants’ circumstances may shift, making periodic renegotiation of roles essential to maintaining alignment and effectiveness [39]. In this context, this toolkit can play a supportive and motivating role by structuring these discussions throughout the project lifecycle, from start to completion, without overloading the project. Nevertheless, it is important to acknowledge that the toolkit is not intended to serve as a strict manual. It requires a certain amount of flexibility and awareness from its users. This is due to its current developmental phase but also to the variability of co-production designs.

One methodological consideration is that the logic for co-production in this work is based on groups tied to projects where partners remain involved over time. This evaluation toolkit is less suited to event-based models, such as those relying on individual workshops where different actors participate at specific points in time. The model emphasizes the value of relational impact, which is challenging to assess in co-production contexts where relationships are not a central element. Thus, further research and improvements need to include the continuous collection of feedback during toolkit use. Following a group from project initiation to post-project completion will allow further development and refinement of the toolkit. Additionally, it would be valuable to explore the toolkit’s potential for evaluating co-production in contexts beyond research. From NSPH and some Swedish national agencies, interest has already been expressed in making the toolkit available for use across several of their collaborative platforms beyond the scope of research. Moreover, it would also be beneficial to study the potential applications of the toolkit outside the mental health sector. Although research reports heightened levels of disempowerment, stigma, and coercion in the mental health contexts, disparities in knowledge validation and power are widely acknowledged as impediments to participation across various domains of care and support [42, 43].

## Conclusions

This study has addressed a critical gap in mental health research by developing a structured, practical, and comprehensive co-production evaluation toolkit. Tailored specifically for mental health contexts, the toolkit provides robust strategies for evaluating both the processes and impacts of co-production throughout a project’s lifecycle. This contribution lays the foundation for the continued development and adoption of co-production approaches, ensuring that mental health research continues to evolve in ways that are inclusive, impactful, and grounded in collaborative principles.

Future research should focus on systematically collecting feedback during the toolkit’s implementation to enhance its usability and optimize its effectiveness. Longitudinal studies that follow co-production research groups from project initiation to post-completion would provide valuable insights for further refinement and validation of the toolkit.

## Electronic supplementary material

Below is the link to the electronic supplementary material.

Supplementary Material 1: GRIPP2 short form

Supplementary Material 2: Face and content validity

Supplementary Material 3: Field-testing

Supplementary Material 4: Initiation guide

Supplementary Material 5: Survey

Supplementary Material 6: Process guide

Supplementary Material 7: Impact guide

## Data Availability

The datasets generated and analysed during the current study are not publicly available due to that individual privacy could be compromised but are available from the corresponding author on reasonable request.

## Abbreviations

EG: Expert group
FTG: Field-tested groups
FG: Field-testing groups
PG: Program group
SU: Service user
SG: Strategy group
NSPH: The Swedish Partnership for Mental Health
WG: Working group
