# Supplementary material for: Development and validation of the UserInvolve comprehensive toolkit for evaluating co-production in research: A guiding resource for researchers
Source: Res Involv Engagem. 2025 Aug 6;11:93. doi: 10.1186/s40900-025-00759-3 (PMC12326713; doi:10.1186/s40900-025-00759-3)
Supplement: Supplementary file 1 — Supplementary Material 1: GRIPP2 short form [file 40900_2025_759_MOESM1_ESM.pdf]

Supplementary Material 1. GRIPP2 short form

Section and topic	Item	Reported on page No
1: Aim	Report the aim of PPI in the study	3-4
2: Methods	Provide a clear description of the methods used for PPI in the study	3-6
3: Study results	Outcomes-Report the results of PPI in the study, including both positive and negative outcomes	6-11
4: Discussion and conclusions	Outcomes-Comment on the extent to which PPI influenced the study overall. Describe positive and negative effects	11-13
5: Reflections/critical perspective	Comment critically on the study, reflecting on the things that went well and those that did not, so others can learn from this experience	12

Staniszewska, S., Brett, J., Simera, I. et al. GRIPP2 reporting checklists: tools to improve reporting of patient and public involvement in research. Res Involv Engagem 3, 13 (2017). https://doi.org/10.1186/s40900-017-0062-2
