# Supplementary material for: Development and validation of the UserInvolve comprehensive toolkit for evaluating co-production in research: A guiding resource for researchers
Source: Res Involv Engagem. 2025 Aug 6;11:93. doi: 10.1186/s40900-025-00759-3 (PMC12326713; doi:10.1186/s40900-025-00759-3)
Supplement: Supplementary file 2 — Supplementary Material 2: Face and content validity [file 40900_2025_759_MOESM2_ESM.pdf]

Supplementary Material 2 - Face and content validity

**SURVEY**	
Comments	Actions
The linkage between survey and group interview is unclear.	Clarify linkage between survey and group interview in information.
The questions under Respect and accessibility “To what extent has time and place for meetings been adjusted according to your needs” and “To what extent have meeting format been adjusted according to your needs” perceived to have an unclear formulation.	Rephrase into bullet list including number of meetings, meeting time, length of meetings, pauses, agenda, meeting place.
Question concerning support, was identified as ambiguous: support from researchers or organizations that partners represent?	Divide into inquiries about support from the "research group" and from the participant's "own organization".
The theme Respect and accessibility: unclear what respect refers to since the questions revolve around accessibility and the capacity to adjust and collaborate within the project.	Rename theme to Accessibility and collaboration.
The theme Representativeness: the questions require the participants to reflect on external conditions rather than internal. Hence, it is not suitable for the survey but important for the group interview.	Remove the theme from survey.
The theme Roles and responsibilities: unclear what responsibilities refers to since the questions are about the possibility to influence.	Rename theme to Roles and opportunity to influence.
The theme Capacity and capability for engagement was perceived as unclear, because it did not effectively convey the factors that facilitate participation in co-production efforts.	Theme was removed and questions were placed under Roles and opportunity to influence and Accessibility and collaboration.
The theme Transparency in communication and documentation: there is no direct question about documentation. Remove theme. The questions fit better under Accessibility and collaboration.	Theme removed and questions placed under Accessibility and collaboration.
The theme Continuity and sustainability: unclear what the theme and its questions refer to. Knowledge and understanding were suggested as a new theme to address potential benefits from the co-produced research process.	Rename theme to Knowledge and understanding, with questions connected to knowledge production and co-production.
More detailed questions about conditions for renumeration was considered relevant.	Too many questions about renumeration would be unproportional considering the wholeness of the survey. Add question about renumeration to the startup guide, as it may be important to discuss for continued participation.
Add more free text questions.	Add free text question at the end of the survey where participants can answer if they have any further input they would like to add.
The questions were ambiguously phrased in relation to the answer options (Likert scale).	Reformulate questions into statements, though statements allow participants to express their level of agreement or disagreement.
**PROCESS-ORIENTED INTERVIEW GUIDE**	
Unclear linkage between survey and group interview.	Clarify linkage between survey and group interview in instructions for moderators.

The interview guide encompassed seven themes: Shared purpose, Respect and acceptability, Representativeness, Roles and responsibilities, Capacity for engagement, Transparency in communication and documentation, and Continuity and sustainability. Adjust themes in accordance with changes in survey themes, except for theme Representation (keep in the interview guide).	Based on input from partners, the interview guide was revised to focus on the following themes: Shared purpose, Roles and opportunity to influence, Representation, Accessibility and collaboration, and Knowledge and understanding.
The need for the interview guide to underscore the importance of understanding participant motivations. The informants identified it as essential to begin by gaining insights into participants' individual motivations and goals for engaging in co-production.	Added as question under Shared purpose: what were your own motives/goals for participating? Have they changed during the project, and if so, how?
Unclear how the Involvement Matrix is going to be used during the interview. Could it be answered individually in advance, added to the survey, or is it going be used as support for stimulating the group interview discussion? How will the data be analyzed (what would, for instance, a scattered result indicates, and how should such a result be dealt with in practical terms within the ongoing research project)?	Test the matrix during the field-testing phase by using it as a discussion-stimulating tool in group interviews. Include guidance for moderators, emphasizing that relevant research phases in the Involvement matrix will be discussed during the interviews.
Some questions in the interview guide repeat what can be discussed when using the Involvement matrix.	Under the theme "Roles and opportunity to influence": Revise the questions to avoid overlapping with the use of the Involvement Matrix during interviews. Ensure the questions complement the matrix, enhancing its effectiveness rather than duplicating its purpose.
Question is needed that allows participants to reflect upon the composition of the group in terms of educational-, socioeconomical-, gendered or ethnic backgrounds.	Add sub-question under the theme Representation:<br>How do you feel individual factors such as gender, age, ethnicity, socio-economic background (where relevant), and contextual factors such as a variation of association, activity, organizations have been considered?
**IMPACT-ORIENTED INTERVIEW GUIDE**	
The word "impact" was used in the guide. It was considered too difficult, since it is brought in from English.	Change to "value" in the Swedish guide (keep “impact” in the English version).
Interview guide structured through the different outcome levels (individual, group/interpersonal, organizational, societal, and paradigmatic) is too vague, abstract and analytical for an interview format.	Reformulate into different kinds of impact for: 1) you 2) group-level 3) organizational level 4) societal level 5) future research and community-based practice.
Unclear what the group-level means: within participants’ project group or in terms of the group participants represent?	Test this during the field-testing phase and understand how participants answer and then decide further action.
The question formulation under the organizational level is complex: How has the co-production been affected by the conditions that your organization works under (for instance statutes, laws, budget, commissions)?	Test the question during field-testing.
Unclear how the Involvement Matrix is going to be used during the interview.	Test this during the field-testing phase by showing the Involvement matrix for stimulating

	discussion during group interview. Then make adjustments if needed.
Add question about any possible personal development or changes in terms of plans for the future under impact on individual/you-level. Such a question could give important information, from all participants, about the individual effects from the co-production.	In what way has participating in the project been significant for you? Has your competence developed? Any other significance? Negative effects?
Add a question to group level about any possible missing competences in the project group.	Question added: have relevant individuals been represented in the co-production? Are there any roles/competence that have been missing? If so, which ones?
