# Supplementary material for: Development and validation of the UserInvolve comprehensive toolkit for evaluating co-production in research: A guiding resource for researchers
Source: Res Involv Engagem. 2025 Aug 6;11:93. doi: 10.1186/s40900-025-00759-3 (PMC12326713; doi:10.1186/s40900-025-00759-3)
Supplement: Supplementary file 3 — Supplementary Material 3: Field-testing [file 40900_2025_759_MOESM3_ESM.pdf]

Supplementary Material 3. Field-testing

EQUAL PARTICIPANTS

**PROJECT INITIATION GUIDE**		**SURVEY**		**PROCESS-ORIENTED INTERVIEW GUIDE**		**IMPACT-ORIENTED INTERVIEW GUIDE**	
**Input**	**Change**	**Input**	**Change**	**Input**	**Change**	**Input**	**Change**
Challenging for researchers not to take a leading role during conversation.	Adjust instructions on how to create a ”we”.	No option to distinguish group of participants (SU-, health care provider/agency representative or researcher)	Add question on participant’s representation (SU: s, health care provider/agency or researcher).	Challenge for researchers to be equal participants. Sometimes researchers tried to stimulate more critical discussions and, by giving examples of own shortcomings, make partners more comfortable with providing criticism.	Add to the instructions for the moderators: the importance of creating an environment in which everyone can be open and willing to provide eventual criticism.	Challenge for researchers to be equal participants. Sometimes researchers tried to stimulate more critical discussions.	Add to the instructions for the moderators: the importance of creating an environment in which everyone can be open and willing to provide eventual criticism.
				Researchers, having initiated the project and taken an active leadership role, found it challenging to respond to the involvement matrix.	Clarify that researchers can participate in the discussion based on what the other participants bring up.		

POWER RELATIONS

PROJECT INITIATION GUIDE		SURVEY		PROCESS-ORIENTED INTERVIEW GUIDE		IMPACT-ORIENTED INTERVIEW GUIDE	
**Input**	**Change**	**Input**	**Change**	**Input**	**Change**	**Input**	**Change**
Partners found it challenging to assert their role in control and decision-making.	Revise the instructions on how to build a cohesive "we" in the co-production process.<br><br>Add the Involvement matrix to start-up guide in to stimulate discussions on how to be involved in the project.			There were concerns about whether participants would feel comfortable expressing critical views if the project leading researchers are included as participants during group interviews.	Enhance the instructions to raise moderators' awareness of the potential impact of the interview format. Additionally, provide partners with an anonymous survey option to ensure a safe avenue for offering critical feedback.	There were concerns about whether participants would feel comfortable expressing critical views if project leading researchers are included as participants during group interviews.	Enhance the instructions to raise moderators' awareness of the potential impact of the interview format. Additionally, provide participants with an anonymous survey option to ensure a safe avenue for offering critical feedback.
At the project initiation, the main goal was to create a secure and confident environment. In some projects, the purpose had been predetermined, leaving some partners	Participants stressed the necessity of continuously revisiting and discussing the research project's purpose with all partners throughout its duration.						

uninvolved in the early stage.							
				An important insight was the significance of the Involvement matrix in stimulating discussions about different levels of co-productions.	Add the Involvement matrix to the start-up guide to ensure alignment with evolving project dynamics.		

BALANCED DOSE

PROJECT INITIATION GUIDE		SURVEY		PROCESS-ORIENTED INTERVIEW GUIDE		IMPACT-ORIENTED INTERVIEW GUIDE	
**Input**	**Change**	**Input**	**Change**	**Input**	**Change**	**Input**	**Change**
Too many and too detailed questions to cover.	Use open themes instead of precise questions.	Survey does not cover enough themes to provide comprehensive understanding about the co-production.<br><br>A relatively large spread of responses on the Likert scale is interpreted as the statements and scale being appropriate.	In the introduction, clarify the aim of the survey, the relevance of the questions and survey's relation to the follow-up group interview.	Too many questions and unclear hierarchy resulted in challenge for moderators to prioritise if running out of time.	Start with a broad entry question and follow-up with possible sub-questions.	Too many sub questions.	Reduce the number of sub-questions.

COMPREHENSIVENESS

PROJECT INITIATION GUIDE		SURVEY		PROCESS-ORIENTED INTERVIEW GUIDE		IMPACT-ORIENTED INTERVIEW GUIDE	
**Input**	**Change**	**Input**	**Change**	**Input**	**Change**	**Input**	**Change**
Researchers felt stressed about requesting partners' time for the project initiation due to the many other project meetings already scheduled.	Clearly articulate the purpose and value of the project initiation meeting to alleviate concerns and emphasize its importance in setting the foundation for collaboration.	Some difficulties in understanding the context of the questions were expressed.	In the introduction, clarify the aim of the survey, the relevance of the questions and survey's relation to the follow-up group interview.	The Involvement matrix was not suitable at the end of the interview, as many of its questions had already been covered during the Roles and opportunity to influence section.	Place Involvement matrix under the theme Roles and opportunity to influence.	The interview guide was not organized in a sufficient way (according to levels of value). It became clear that participants often addressed multiple levels of value in a single answer, making it difficult for the moderator to effectively lead the interviews.	Revise the interview guide to focus on new themes (relational, instrumental, conceptual), allowing the content to shape the questions and incorporating multiple levels of value within each question. This new structure in the interview guide was tested with good results.
				The order of the themes was not appropriate. Theme Roles & opportunity to influence fits better after Shared purpose.	Change order of the themes according to the input.		

				Accessibility & collaboration fits better after Representation.			

FLEXIBILITY

PROJECT INITIATION GUIDE		SURVEY		PROCESS-ORIENTED INTERVIEW GUIDE		IMPACT-ORIENTED INTERVIEW GUIDE	
**Input**	**Change**	**Input**	**Change**	**Input**	**Change**	**Input**	**Change**
The questions were framed as interview questions rather than open discussion themes.	Shift to using broader themes to foster dialogue instead of precise questions. This approach aligns better with the co-production format, especially at the project initiation, where it's crucial to have an open conversation about how participants wish to collaborate.	The option to add free text answers was requested for some questions.	Add a free text question in the end of the survey. Further, the group interview provides an opportunity to develop answers.	Some questions may appear to be more relevant to partners than researchers.	Encourage researchers to reflect on them as well, ensuring they are inclusive of both perspectives.	Some questions were unclear and difficult to understand.	Go through and reformulate questions to enable more flexible format.
