# Supplementary material for: Development and validation of the UserInvolve comprehensive toolkit for evaluating co-production in research: A guiding resource for researchers
Source: Res Involv Engagem. 2025 Aug 6;11:93. doi: 10.1186/s40900-025-00759-3 (PMC12326713; doi:10.1186/s40900-025-00759-3)
Supplement: Supplementary file 4 — Supplementary Material 4: Initiation guide [file 40900_2025_759_MOESM4_ESM.pdf]

UserInvolve's evaluation toolkit for research through co-production

Guide for project initiation

Purpose and goal of the group interview

This conversation guide can be used to stimulate discussions between researchers and partners about the upcoming co-production. It can help create a sense of group cohesion and establish a common "we".

Setup

The guide is designed to support an open and creative dialogue between all participants and not therefore as an interview guide to collect baseline data based on answering questions. During the conversation, researchers can switch between presenting the project, if a project plan already exists, and actively encouraging partners to ask questions and share their thoughts. This not only contributes to a rewarding discussion but also provides valuable insights from partners who then can influence and improve the project's decisions and direction. By creating an open dialogue, researchers and partners can shape the project together, which strengthens the sense of involvement and joint responsibility.

Introduction

The conversation is initiated by the project leader for the research project by:

- Presenting the purpose of the conversation
- The project leader explaining what co-production means and why it is important in the project/work
- The project leader asking everyone to introduce themselves and share who they represent in the research project

Conversation questions

The conversation then focuses on how to work in co-production and what expectations and thoughts everyone has about participating.

1. **The project leader initiates a conversation about the purpose of the project.**
   If the purpose is already established, the project leader can describe it, and then move on to a discussion about, for example, how the group views the project's purpose and goals, their own motivations for participating, and how the research project is important for the target groups/the organization partners represent.

2. **The project leader initiates a conversation about accessibility and collaboration.**

    - Are there any needs regarding the number of meetings, meeting time, length of meetings, breaks, meeting order, and meeting location?
    - How can a working climate be created that makes everyone feel comfortable sharing their perspective, for example, to speak up if they have a differing opinion or do not understand.
    - What support can the project leading researchers provide for everyone to participate in the project? Do the partners need any support from their respective organizations to participate?
    - What are your thoughts on resources concerning your participation?
    - What can be done to make participation feel meaningful?

3. **The project leader initiates a conversation about representation.**

    The conversation can, for example, address whether the necessary competences have been included in the project group to achieve the project's purpose and goals or if there are other people who should be invited.

4. **The project leader initiates a conversation about roles and the opportunity to influence.**

    The conversation about roles can be based on the UserInvolve version of the Involvement matrix (see at the end of the guide). It involves a dialogue together about how much one wishes to be involved in the project, how much time can be allocated in the different phases, and in which phases one sees their competence best utilized in the project. It can end with a conversation about how transparency and feedback can be achieved in the project (for example, how information, decisions, etc. are shared between each other).

5. **The project leader initiates a conversation about knowledge and understanding.**

    The conversation concerns everyone's expectations for the upcoming co-production and what they hope it will lead to in terms of values, knowledge, and outcomes, both in relation to the project and in relation to their own organization. For example, what do they hope that participation will lead to in their own organization?

6. **The project leader concludes the conversation**
    This is done by summarizing what has been discussed and providing information about what will happen next. It is good to ask if anyone has anything more to add or ask.

UserInvolve's version of Involvement matrix[1]

ROLE IN PROJECT/RESEARCH

STAGES OF PROJECT/RESEARCH		Listener<br>Is given information	Co-thinker<br>Is asked to give opinion	Advisor<br>Gives (un)solicited advice	Partner<br>Works as an equal partner	Decision-maker<br>Takes initiative, (final) decision
Preparation	Influence in the identification and prioritization of research needs.					
	Influence in defining research questions.					
	Influence in designing the project.					
	Influence over the funding application					
	Influence over the ethics application.					
Execution	Influence over data collection.					
	Influence in data analysis.					
Communication of results	Influence in the writing of articles.					
	Influence in the writing of reports.					
	Influence in other reporting activities.					
Implemen-tation	Influence in the application of results in practice.					

[1] Smits DW, Van Meeteren K, Klem M, Alsem M, Ketelaar M. Designing a tool to support patient and public involvement in research projects: The Involvement Matrix. Research involvement and engagement. 2020;6(1):30-7.
