# Supplementary material for: Development and validation of the UserInvolve comprehensive toolkit for evaluating co-production in research: A guiding resource for researchers
Source: Res Involv Engagem. 2025 Aug 6;11:93. doi: 10.1186/s40900-025-00759-3 (PMC12326713; doi:10.1186/s40900-025-00759-3)
Supplement: Supplementary file 5 — Supplementary Material 5: Survey [file 40900_2025_759_MOESM5_ESM.pdf]

UserInvolve's evaluation toolkit for research through co-production

Process-oriented survey

Study ID: _______________

The survey concerns the following co-production group/project:

- ○ Choice a
- ○ Choice b
- ○ Choice c
- ○ Etc.

In the co-production group/project my primary role is to represent:

- ○ Service user, patient, family carer
- ○ Health or care provider/agency
- ○ Researcher
- ○ Other

What phase is the project in? ○ Mid-point ○ Project completion/post project

**Theme: Shared purpose**	Strongly Disagree	Disagree	Somewhat Disagree	Somewhat Agree	Agree	Strongly Agree	Cannot Assess
I know the purpose of the research project.	○	○	○	○	○	○	○
I have been part of formulating the purpose.	○	○	○	○	○	○	○
I think the purpose is meaningful.	○	○	○	○	○	○	○

Theme: Accessibility and collaboration

The working methods have suited me regarding:

	Strongly Disagree	Disagree	Somewhat Disagree	Somewhat Agree	Agree	Strongly Agree	Cannot Assess
• number of meetings	○	○	○	○	○	○	○
• time of meetings	○	○	○	○	○	○	○
• length of meetings	○	○	○	○	○	○	○
• breaks	○	○	○	○	○	○	○
• agenda of meetings	○	○	○	○	○	○	○
• meeting location	○	○	○	○	○	○	○

**Theme: Accessibility och cooperation**	Strongly Disagree	Disagree	Somewhat Disagree	Somewhat Agree	Agree	Strongly Agree	Cannot Assess
I have been able to influence the working methods.	○	○	○	○	○	○	○
It has been easy to communicate with others in the project.	○	○	○	○	○	○	○
The communication has been clear about ongoing research and results.	○	○	○	○	○	○	○
I have felt safe expressing my thoughts about the project.	○	○	○	○	○	○	○
I have had enough time to contribute to the project.	○	○	○	○	○	○	○
I have received the support from the research group that I might have needed to participate in the project.	○	○	○	○	○	○	○
I have received the support from my own organization that I might have needed to participate in the project.	○	○	○	○	○	○	○
The possible financial compensation has been reasonable.	○	○	○	○	○	○	○

**Theme: Roles and opportunity to influence**	Strongly Disagree	Disagree	Somewhat Disagree	Somewhat Agree	Agree	Strongly Agree	Cannot Assess
My role in the research project has been clear for me.	○	○	○	○	○	○	○
I have been able to influence my role in the project.	○	○	○	○	○	○	○
I have been able to influence decision making in the project.	○	○	○	○	○	○	○
My expertise has been utilized in the project.	○	○	○	○	○	○	○
I have received feedback on how my contributions have been utilized in the project.	○	○	○	○	○	○	○
I have felt like a valuable participant in the project.	○	○	○	○	○	○	○

**Theme: Knowledge and understanding**	Strongly Disagree	Disagree	Somewhat Disagree	Somewhat Agree	Agree	Strongly Agree	Cannot Assess
My knowledge about the subject of the project has increased.	○	○	○	○	○	○	○
My understanding of the other stakeholders involved in the project has increased.	○	○	○	○	○	○	○
I see an advantage in working through co-production.	○	○	○	○	○	○	○

Is there anything else you would like to convey to us?

If so, please write below
