# Supplementary material for: Development and validation of the UserInvolve comprehensive toolkit for evaluating co-production in research: A guiding resource for researchers
Source: Res Involv Engagem. 2025 Aug 6;11:93. doi: 10.1186/s40900-025-00759-3 (PMC12326713; doi:10.1186/s40900-025-00759-3)
Supplement: Supplementary file 6 — Supplementary Material 6: Process guide [file 40900_2025_759_MOESM6_ESM.pdf]

UserInvolve’s evaluation toolkit for research through co-production

Process-oriented interview guide at project´s mid-point

Purpose and goal of the group interview

The interview guide can be used at the mid-point of a research project to capture experiences of the co-production process to evaluate how it can be improved during the remaining project time.

Setup

The group interview gathers all involved project participants (researchers and partners). Two moderators, who have not been actively engaged in the project, lead the discussion. The moderators come from other parts of the research program, related research projects, or, if needed, from an external context. It is welcome if one of the moderators wants to be open about having lived experience of mental health challenges. This may contribute to a safe and inclusive discussion environment for discussion. All co-production partners and researchers participate, reflecting from their own perspectives regardless of their role in the project.

Introduction

At the beginning, the moderators introduce themselves and inform the participants about the purpose of the interview:

- **Learning from various projects.** We strive to learn from our co-production projects with user movements, regions, municipalities, and other organizations. Our research should be relevant and valuable to those it concerns. Therefore, we want to understand and learn more from this project.
- **Purpose of the interview**. We want to learn how your experience of co-production has been in the project so far. The intention is that this conversation will also be valuable for you in the project and provide an opportunity for reflection on what has worked well and possible changes or improvements that can be made for the remaining project time.
- **Connection to the survey.** The themes in the survey and the group interview largely overlap, but the survey emphasizes the possibility of responding individually and anonymously. The group interview provides an opportunity for in-depth collective conversation.
- **Open discussion environment.** There are no right or wrong answers (it is important to create as open and permissive a discussion climate as possible).
- **Researchers as participants.** Researchers participate in the meeting in the same way as co-production partners.

After this introduction, the moderators ask everyone to briefly introduce themselves by stating their name and who they represent in the co-production group.

Interview Questions

Shared purpose

Open question: How would you describe your own motives and goals for participating in the project?

Possible follow-up questions:

- How do you view the project's purpose and goals?
- How have you had the opportunity to influence it?
- In what way is the research project important for the target groups/organization you represent?

Roles and opportunity to influence

Open question: How have you experienced your different roles in the project? The dialogue can be guided by UserInvolve's version of the Involvement matrix (see at the end of the guide):

The moderators show and describes the matrix to the participants. The matrix covers influence during the planning phase and the implementation phase of the research project, and the group discusses this theme (i.e., roles and opportunity to influence) in the relevant phase(s). Here, researchers can participate in the discussion based on what the other participants bring up.

Possible follow-up questions:

- What roles do you feel you have been given or taken in the project? Would you like your roles to evolve, and if so, how?
- How do you feel your knowledge and competence have been utilized in the project? Does anything need to change to improve this?
- Have you received feedback on how your contributions have been utilized in the project? If so, in what ways?

Representation

Open question: How do you feel different knowledge and experiences have been included in the project?

Possible follow-up questions:

- How do you feel individual factors such as gender, age, ethnicity, socio-economic background (where relevant), and contextual factors such as a variation of association, activity, organizations have been considered?
- Are there groups affected by the research that should have been included to a greater extent? Which ones, if any?

Accessibility and collaboration

Open question: How have you experienced participating in meetings and collaborating within the project?

Possible follow-up questions:

- How have the meeting formats worked for you? For example, regarding the number of meetings, meeting time, length of meetings, breaks, meeting order, and meeting location?
- How have you experienced the accessibility of information?
- How has it been to communicate in the project? Has it felt safe to express differing opinions or ask if you did not understand something?
- Have you received the support you needed to participate in the project? Both from the project and from your own organizations?
- What has been important for you to feel valuable as a participant in the project? How can this be developed?

Knowledge and understanding

Open question: How has your understanding and knowledge developed through your participation in the project?

Possible follow-up questions:

- Has your knowledge of co-production increased through the way you have worked? If so, how?
- Do you feel your knowledge of each other and your organizations has increased through the co-production process? In what way?
- Have you discovered other impact through participating in the work that you did not expect from the beginning?

The interview concludes with the moderators:

- Asking if anyone wants to add anything to what has been said.
- Asking the participants if the meeting has given them any new insights, and if so, what they are.
- Summarizing what has emerged regarding obstacles and enabling factors for a successful continuation of the co-production process.

UserInvolve's version of Involvement matrix[1]

STAGES OF PROJECT/RESEARCH — ROLE IN PROJECT/RESEARCH

Stage		Listener: Is given information	Co-thinker: Is asked to give opinion	Advisor: Gives (un)solicited advice	Partner: Works as an equal partner	Decision-maker: Takes initiative, (final) decision
Preparation	Influence in the identification and prioritization of research needs.					
	Influence in defining research questions.					
	Influence in designing the project.					
	Influence over the funding application					
	Influence over the ethics application.					
Execution	Influence over data collection.					
	Influence in data analysis.					
Communication of results	Influence in the writing of articles.					
	Influence in the writing of reports.					
	Influence in other reporting activities.					
Implementation	Influence in the application of results in practice.					

[1] Smits DW, Van Meeteren K, Klem M, Alsem M, Ketelaar M. Designing a tool to support patient and public involvement in research projects: The Involvement Matrix. Research involvement and engagement. 2020;6(1):30-7.
