# Supplementary material for: Development and validation of the UserInvolve comprehensive toolkit for evaluating co-production in research: A guiding resource for researchers
Source: Res Involv Engagem. 2025 Aug 6;11:93. doi: 10.1186/s40900-025-00759-3 (PMC12326713; doi:10.1186/s40900-025-00759-3)
Supplement: Supplementary file 7 — Supplementary Material 7: Impact guide [file 40900_2025_759_MOESM7_ESM.pdf]

UserInvolve's evaluation package for research through co-production

Impact-oriented interview guide, post-project

Purpose and goal of the group interview

This interview guide can be used at the end of the project to support researchers and their partners in evaluating the co-production in the project. The guide can help to stimulate conversations between researchers and partners about how and what the co-production has led to in terms of value, knowledge, and benefit. It is focused on the effects and results that the participants believe can be attributed to the co-production. The questions are based on the level of change – i.e., the changes that have occurred at the individual level, organization/activity level, and societal/structural level.

Setup

The group interview gathers all involved participants (researchers and partners). Two moderators, who have not been actively engaged in the project, lead the discussion. The moderators come from other parts of the research program, related research projects or, if needed, from an external context. It is welcome if one of the moderators wants to be open about their lived experience with mental health challenges. This may contribute to a safe and inclusive environment for discussion. All co-production partners and researchers participate, reflecting from their own perspectives, regardless of their role in the project.

Introduction

At the beginning, the moderators introduce themselves and inform the participants about the purpose of the interview:

- **Learning from various projects.** We strive to learn from our co-production projects with user movements, regions, municipalities, and other organizations. Our research should be relevant and valuable to those it concerns. Therefore, we want to understand and learn more from this project.
- **Purpose of the interview.** We want to learn more about what your participation in this co-production project has led to in terms of impact. You have previously had the opportunity to respond to a survey and participate in a group interview about how you experienced the co-produced research process. This group interview aims instead to investigate whether and how the co-production has led to any impact, both individually for you and your organization. We also want to understand what has worked well and what have been the obstacles to success.
- **Open discussion environment.** There are no right or wrong answers (it is important to create as open and permissive a discussion climate as possible).
- **Researchers as participants.** Researchers participate in the meeting as participants in the same way as co-production partners.

After this introduction, the moderators ask everyone to briefly introduce themselves by stating their name and who they represent in the co-production group.

Interview questions

Roles and opportunity to influence

The interview starts by following up on the roles the participants have had during the project's implementation.

Open question: What roles do you feel you have been given or taken in the project? Have they changed during the project?

Concrete changes

(changes regarding what has happened in terms of plans, decisions, behaviors, practices, and policies. It can also be about changes in skills and expertise).

Open question: Do you have examples of concrete changes that have been achieved through the project?

Possible follow-up questions:

- Has your way of working changed due to your participation in the project? If so, how?
- Has participation in the project contributed to new ways of working for your organization? Due to co-production? Due to the research results?
- Has participation in the project contributed to new ways of researching in co-production?
- Has the project led to opportunities to influence politics and policy development?

Knowledge and perspective changes

(changes regarding knowledge, awareness, attitudes, and perspectives).

Open question: How would you describe any changes in your knowledge, awareness, or perspectives that your involvement in the project has resulted in?

Possible follow-up questions:

- In what way has participation in the project contributed to knowledge, competence, and new perspectives for you?
- Has the project contributed to new knowledge/competence/new perspectives for your organization?
- Has the project contributed to developing new knowledge/theories and concepts? What factors have enabled this contribution?

Relational changes

(changes in the number of relationships and the quality of relationships and cooperation).

Open question: How have your relationships been affected by participation in the project?

Possible follow-up questions:

- What significance has it had for you to meet the others in the group?
- Do you feel that the collaboration between the various participating organizations/activities has been affected outside the project?
- Have new networks or collaborations been created?

Process development

Open question: What lessons about co-production would you like to pass on for future projects?

Possible follow-up questions:

- What is important to consider for co-production to be meaningful for those involved?
- What is important for co-production to have an impact for the organizations involved?
- What obstacles and opportunities for co-production have you seen that are valuable to highlight?
- What conditions are important for co-production to lead to change on a broader societal level?

The interview concludes with the moderators:

- Asking if anyone wants to add anything to what has been said.
- Asking the participants if the meeting has given them any new insights, and if so, what they are.
